# Optimizing the Design of TES Tanks for Thermal Energy Storage Applications Through an Integrated Biomimetic-Genetic Algorithm Approach

**DOI:** 10.3390/biomimetics10040197

**Published:** 2025-03-24

**Authors:** Nadiya Mehraj, Carles Mateu, Gabriel Zsembinszki, Luisa F. Cabeza

**Affiliations:** GREiA Research Group, Universitat de Lleida, Pere de Cabrera s/n, 25001 Lleida, Spain; nadiya.mehraj@udl.cat (N.M.); gabriel.zsembinszki@udl.cat (G.Z.)

**Keywords:** thermal energy storage (TES), bio-inspired TES tank, biomimetics, genetic algorithm (GA), design optimization, artificial intelligence (AI)

## Abstract

Building upon an experimentally validated bio-inspired thermal energy storage (TES) tank design, this study introduced a novel computational framework that integrated genetic algorithms (GA) with biomimetic principles to systematically generate TES tank geometries. Inspired by natural thermal distribution patterns found in vascular networks, the AI-driven methodology explored 13 geometric parameters, focusing on branching structures and spatial distribution, and resulted in computationally generated designs with a 29% increase in heat transfer surface area while maintaining manufacturability constraints within a fixed tank diameter of 150 mm and height of 155 mm. Unlike previous biomimetic TES studies that relied on predefined geometric configurations, this approach developed AI-driven bio-inspired structures within experimentally validated dimensional constraints, ensuring geometric relevance while allowing for broader structural exploration. The resulting designs exhibited key characteristics of high-efficiency bio-inspired configurations while providing a systematic, scalable methodology for TES tank architecture. This study represented the first step in integrating AI-driven biomimicry into TES tank design, establishing a structured framework for generating high-performance, manufacturable configurations. While the current work focused on computational design, future research will emphasize experimental validation and real-world implementation to confirm the practical thermal and structural benefits of these AI-generated bio-inspired designs. By bridging the gap between computational intelligence and nature-inspired engineering, this research provided a scalable pathway for developing more efficient, manufacturable, and sustainable TES solutions for energy storage applications.

## 1. Introduction

Thermal energy storage (TES) tanks emerged as a cornerstone technology in advancing sustainable energy solutions, with recent studies demonstrating their capacity to improve system efficiency by 20–40% across diverse applications [1,2]. The integration of TES systems became increasingly vital in addressing renewable energy intermittency, reducing peak load fluctuations by up to 50% while enhancing overall energy efficiency through improved thermal management capabilities [3,4]. The effectiveness of these systems fundamentally depended on their design architecture, particularly in latent thermal storage applications where phase change materials (PCMs) served as the primary storage medium, offering energy densities 5–14 times higher than sensible heat storage systems [5,6].

Traditional TES tank designs, however, faced significant performance limitations, with heat transfer rates typically constrained to 15–30% of their theoretical potential due to insufficient surface area and thermal conductivity barriers [7,8]. These inherent limitations substantially impacted charging and discharging rates, with conventional designs requiring charging cycles that were 2–3 times longer compared to theoretical optimums [9,10]. A recent experimental study by Cabeza et al. (2024) [11] demonstrated the effectiveness of a bio-inspired TES tank design that achieved 52% faster discharge rates compared to conventional shell-and-tube configurations. Their design, mimicking biological vascular networks, provided a promising foundation for further optimization. While their work established the viability of biomimetic principles in TES tank design, the geometric parameters were not systematically optimized, presenting an opportunity for significant performance enhancement through computational optimization techniques.

The evolution of TES tank optimization has accelerated significantly through artificial intelligence (AI) integration, particularly genetic algorithms (GA) [12,13]. These evolutionary computation methods demonstrated up to 35% improvement in thermal system optimization compared to traditional approaches [14,15]. GA excelled in handling complex design spaces with multiple variables, consistently outperforming conventional optimization methods by 25–40% while effectively avoiding local optima [16,17].

Nature sophisticated thermal management solutions, particularly evident in the branching patterns of trees and vascular networks, achieved remarkable efficiency through evolutionary refinement. These biological systems demonstrate thermal distribution efficiencies of up to 90% of the theoretical limits while minimizing material usage by 30–50% compared to conventional engineered systems [18,19,20,21]. The strategic application of such biomimetic principles to TES tank design offered promising pathways for performance enhancement, with preliminary studies showing potential efficiency improvements of 25–60% [22,23].

Despite these advances, a significant research gap existed in systematically combining biomimetic principles with optimization algorithms. While isolated studies of bio-inspired TES designs showed promising results [24,25], comprehensive frameworks integrating evolutionary optimization with biomimetic design principles remained largely unexplored [26,27]. The synergistic combination of these approaches presented an unprecedented opportunity to revolutionize TES tank design, potentially offering efficiency improvements of 30–70% compared to conventional approaches [14,15].

Although AI-driven optimization has increasingly integrated into biomimetic design across diverse engineering fields, its application in TES tank optimization remained largely unexplored. Previous studies demonstrated the effectiveness of GA-optimized biomimetic design in structural engineering, aerodynamics, robotics, and materials science, highlighting its potential to enhance performance and efficiency. However, no prior work has systematically applied this approach to TES tank design. This study addressed this gap by integrating genetic algorithms with biomimetic principles to develop a scalable and computationally optimized framework for TES system enhancement.

To contextualize the novelty of this study, Table 1 presents a summary of existing GA-biomimetic applications across various fields, highlighting the research gap in TES tank optimization.

Previous studies on TES tank design optimization primarily focused on conventional geometries and parametric modifications of existing configurations [4]. While bio-inspired designs showed promise in experimental testing, these designs were developed without systematic consideration of the underlying physical principles or optimization of their geometrical features. The initial bio-inspired TES tank prototype demonstrated enhanced discharge performance compared to traditional shell-and-tube configurations, yet its design parameters were not optimized [11].

This work advanced TES tank design through the following:

(a) Development of a GA optimization framework to enhance the performance of an experimentally validated bio-inspired TES tank design;
(b) Systematic optimization of surface area through branching structure evolution, maximizing thermal performance without modifying the basic bio-inspired concept; and
(c) Quantitative assessment of GA optimization benefits for bio-inspired TES tank configurations.

Uniquely, this work bridged the gap between experimental validation (Cabeza et al. (2024)) [11] and design optimization by applying GA to an existing successful bio-inspired design. Rather than fundamentally altering the design concept, the optimization framework focused specifically on maximizing heat transfer surface area while maintaining the core biomimetic principles that proved effective in experimental testing. The results provided concrete guidelines for improving bio-inspired TES tank performance through targeted geometrical optimization, enabling practical applications in sustainable energy systems such as waste heat recovery and solar thermal storage.

The primary objective of this study was to establish a systematic methodology for optimizing a bio-inspired TES tank design using GA. This work aimed to demonstrate the integration of AI-driven optimization with biomimetic principles, focusing on enhancing thermal performance while ensuring manufacturability. Rather than solely emphasizing surface area enhancement, the study bridged the gap between experimental validation and computational optimization, showcasing a robust framework for advancing sustainable energy systems.

## 2. Literature Review

Recent advances in TES technology pushed the boundaries of energy storage capabilities, achieving energy density improvements of 30–45% through innovative materials and system designs [35]. Despite significant progress in optimizing TES designs through material and geometric modifications, no study had systematically combined AI-driven biomimetic methodologies to generate TES tank structures. While prior works explored pre-defined bio-inspired geometries, they did not integrate computational intelligence to autonomously generate and refine complex branching structures. This study addressed this gap by developing an AI-driven generative framework that mimicked natural vascular networks while ensuring manufacturability constraints, laying the foundation for a more scalable approach to TES design. Recent comprehensive reviews by Cabeza et al. (2021) [3] and Jouhara et al. (2020) [36] established the fundamentals of TES systems, highlighting developments in materials and system integration that achieved thermal conductivity enhancements of up to 85%. Sadeghi (2022) [37] further emphasized the critical challenges in thermal storage development, documenting efficiency losses of 20–35% due to suboptimal design integration and material selection.

The design optimization of TES tanks showed significant potential for performance enhancement, with recent studies reporting efficiency improvements of 25–60% through innovative geometrical configurations. Yang et al. (2021) [22] developed a systematic methodology for a shell-and-tube latent thermal energy storage design that achieved a 40% reduction in charging time through optimized material selection and geometry. Bouchenna et al. (2021) [38] demonstrated that optimized tank configurations could enhance heat transfer rates by up to 55% compared to conventional designs, while reducing material usage by 15–30%. These advancements in design optimization established a clear correlation between geometric configuration and thermal performance, with surface area improvements directly translating to enhanced heat transfer capabilities.

Recent advancements in biomimetic principles introduced a new dimension to TES design. Omidvarnia et al. (2024) [25] applied vascular-inspired geometries to enhance thermal distribution, while Zhang et al. (2023) [39] demonstrated improved performance using biomimetic oval geometries. However, these studies primarily relied on static bio-inspired geometries without systematic computational variation. The approach of this study advanced this research by introducing AI-driven generative design, where genetic algorithms dynamically created bio-inspired TES structures, rather than relying on pre-defined templates. This computational framework allowed for a more adaptive and scalable approach to TES tank development, ensuring manufacturability while systematically enhancing surface area [39]. Mohtasim and Das (2024) [40] explored bio-derived and biomimetic composite PCMs for TES applications, achieving substantial increases in thermal conductivity. Furthermore, Huang et al. (2023) [41] investigated innovative biomimetic fin designs for heat recovery in data centers, highlighting their ability to enhance phase-change heat transfer processes. However, none of these approaches systematically combined AI-driven optimization with bio-inspired geometries, as this study does. This study uniquely integrated genetic algorithms with biomimetic principles, achieving a significant 29% improvement in surface area while adhering to practical manufacturing constraints, thereby addressing a critical gap in existing methodologies.

Also, Liu et al. (2024) [42] demonstrated novel PCM compositions achieving energy density improvements of up to 52% through optimized molecular structures while Kumar et al. (2023) [43] reported breakthrough developments in nano-enhanced PCMs, documenting thermal conductivity improvements of up to 127% through systematic material engineering approaches. Recent advancements in thermochromic microencapsulated phase change materials (PCMs) led to significant improvements in thermal energy absorption and storage efficiency. For instance, a study by Zhou et al. (2024) [44] developed leak-proof reversible thermochromic microcapsule PCMs with high latent heat storage capacity and excellent thermal stability. While these material-based approaches provided crucial enhancements, they operated independently of the structural design of thermal energy storage (TES) tanks. This study differentiated itself by focusing on the physical architecture of TES tanks, leveraging AI-driven biomimicry to systematically generate and refine bio-inspired geometries.

Meanwhile, recent advancements in optimization techniques significantly contributed to the development of TES systems and TES tank designs. While stochastic optimization techniques were extensively explored for hybrid energy storage integration, as demonstrated by Garcia-Torres et al. (2021) [45], these approaches primarily focused on economic and operational strategies rather than the structural design of storage systems. This study differentiated itself by focusing on the physical architecture of thermal energy storage (TES) tanks, leveraging AI-driven biomimicry to systematically generate and refine bio-inspired geometries. Also, topology optimization was widely adopted to enhance the structural and functional efficiency of heat exchangers by optimizing material distribution, as reviewed by Sigmund et al. (2013) [46]. Additionally, multi-fidelity optimization frameworks, such as those explored by Fernández-Godino et al. (2016) [47] offered a balance between computational cost and model accuracy, making them highly effective for complex engineering systems. These approaches showed success in optimizing TES designs, particularly under computational constraints.

The advanced methods, including deep learning and reinforcement learning by Thuerey et al. (2020) [48] and Rahmani et al. (2024) [49], demonstrated potential in optimizing thermal and fluid dynamics, yet their integration with biomimetic principles remained largely unexplored. The current study uniquely addressed this gap by combining genetic algorithms with biomimetic principles, leveraging the strengths of evolutionary optimization to achieve practical and manufacturable designs. Future research could further build upon this work by integrating multi-fidelity and machine learning-based optimization approaches to develop more efficient TES systems.

Biomimetic approaches revolutionized TES tank design, with nature-inspired configurations demonstrating remarkable efficiency gains. Recent studies by Jamei et al. (2021) [50] documented energy efficiency enhancements of up to 45% in building applications. Zhang et al. (2023) [39] specifically examined bionic approaches to latent heat thermal storage, demonstrating heat transfer improvements of 40–60% through bio-inspired geometries. These biological systems, refined through evolutionary processes, consistently outperformed traditional engineered solutions, with some designs achieving up to 80% of theoretical performance limits while minimizing material usage. The practical implementation of biomimetic principles in TES tank design was recently demonstrated by Cabeza et al. (2024) [11] who developed and tested a bio-inspired branching structure that increased heat transfer surface area without requiring additional enhancement methods like fins. Their experimental results showed significant improvements in discharge performance, establishing a baseline for further optimization studies. Mao (2020) [51] demonstrated leaf-venation-inspired cooling systems with 55% improved thermal distribution uniformity. These recent developments highlight the continuing evolution of biomimetic approaches in thermal system design.

While biomimetic designs demonstrated substantial efficiency gains in TES applications, their integration into manufacturable systems remains a key challenge. Previous studies primarily explored predefined bio-inspired structures, lacking a systematic method to evaluate manufacturability constraints. The AI-driven biomimetic framework presented in this study addressed this gap by autonomously generating and refining TES tank designs within experimentally validated geometric ranges. By ensuring that the produced configurations resemble the tested designs of Cabeza et al. (2024) [11], this approach bridged the gap between computational generation and real-world implementation, facilitating direct translation into additive manufacturing and large-scale energy storage applications. Future work will focus on validating these computationally generated models through experimental testing, confirming their performance under practical conditions.

The integration of AI, particularly GA, has emerged as a powerful tool for TES tank optimization. Olabi et al. (2023) [52] demonstrated performance improvements of 25–40% through AI-optimized thermal storage systems, while Wang and Zhang [53,54] achieved energy efficiency gains of 30–45% through dynamic optimization. Chen et al. (2023) [55] applied hybrid intelligence approaches that balanced multiple performance objectives, resulting in overall system improvements of 35–55%. These studies established GAs as particularly effective in handling complex design optimization problems, consistently outperforming traditional optimization methods by 20–30%. Recent applications of GAs in TES showed impressive results. Li et al. (2024) [56] achieved a 58% performance improvement through the multi-objective GA optimization of shell-and-tube heat exchangers. Farahani et al. (2024) [57] demonstrated the effectiveness of hybrid GA-neural network approaches, achieving 43% better convergence rates in TES system optimization while maintaining solution quality.

Recent advancements in TES optimization introduced hybrid and multi-objective techniques that significantly enhance system performance. For instance, Xu et al. (2024) [58] employed a hybrid method combining the non-dominated sorting genetic algorithm II (NSGA-II) with invasive weed optimization (IWO) to optimize fan duct surface heat exchanger configurations, achieving a 25% improvement in thermal performance. Similarly, Shokouhmand et al. (2020) [59] explored a multi-objective optimization of plate-fin heat exchangers, focusing on minimizing flow maldistribution and maximizing thermal efficiency. Makhadmeh et al. (2022) [60] reviewed advancements in hybrid algorithms for thermal energy systems, emphasizing their ability to address complex design constraints. Moon et al. (2021) [61] developed ultra-power-dense heat exchangers using a genetic algorithm integrated with metal additive manufacturing, achieving a 203% higher specific power compared to conventional designs, demonstrating the potential of combining genetic algorithms with advanced manufacturing techniques. Colaço et al. (2022) [62] utilized a reinforcement learning-based genetic algorithm to optimize double-pipe heat exchangers with perforated baffles, improving the thermal performance index by 78% while addressing constraints in geometric design. These advancements highlighted the need for hybrid approaches in TES and related fields, motivating the integration of genetic algorithms with biomimetic principles in this study.

Despite advances in TES design, existing studies often lacked a systematic optimization of the bio-inspired geometries, focusing instead on isolated parameters or application-specific configurations. While biomimetic principles demonstrated potential, their integration with AI-driven techniques, such as genetic algorithms, remained underexplored. This study uniquely addressed these gaps by systematically combining biomimetic design principles with genetic algorithm optimization, offering a framework for improving TES tank performance while ensuring practical manufacturability.

Hankins and Fertig [63] documented potential performance improvements of 40–70% through combined bio-inspired and algorithmic optimization approaches. While various optimization strategies have been applied to TES, including tube layout optimization which achieved 25–35% improvement and flow arrangement optimizations which yielded 30–45% enhancement rates [53,54,55], the systematic combination of biomimetic principles with evolutionary algorithms remains underdeveloped. This research gap suggested significant potential for enhancing TES tank performance through the synergistic application of nature-inspired design principles and advanced optimization techniques [26,63], particularly in developing comprehensive frameworks that can simultaneously optimize multiple performance parameters while maintaining practical manufacturing constraints.

Unlike previous biomimetic TES studies that relied on predefined geometric structures (Omidvarnia et al., 2024, Zhang et al., 2023) [25,39], this study introduced a computational framework that autonomously generates optimized TES tank geometries using AI. Prior works demonstrated the effectiveness of biomimetic forms for improving heat transfer, but they lacked a systematic approach for iteratively refining and validating these designs within practical constraints. The AI-driven methodology introduced in this study ensured that the generated designs not only exhibited enhanced thermal efficiency but also adhered to manufacturability limits, allowing for a seamless transition to prototyping and experimental validation in future research.

To consolidate the findings and provide a clear understanding of the existing research landscape, Table 2 summarizes the key studies on TES optimization, biomimetic principles, and hybrid algorithms. This table highlighted the contributions of recent advancements and underscored the research gaps addressed by the proposed study.

## 3. Methodology

This study built upon the successful bio-inspired TES tank design developed by Cabeza et al. (2024) [11], aiming to optimize its geometric parameters through genetic algorithms. The branching structure of the original design, which demonstrated superior discharge performance, provided the fundamental architecture for this optimization framework. The manufacturing constraints and dimensional boundaries established in their experimental validation directly informed these optimization parameters, ensuring the theoretical improvements remain practically feasible.

### 3.1. Bio-Inspired TES Tank Design Concept

**Figure 1:**
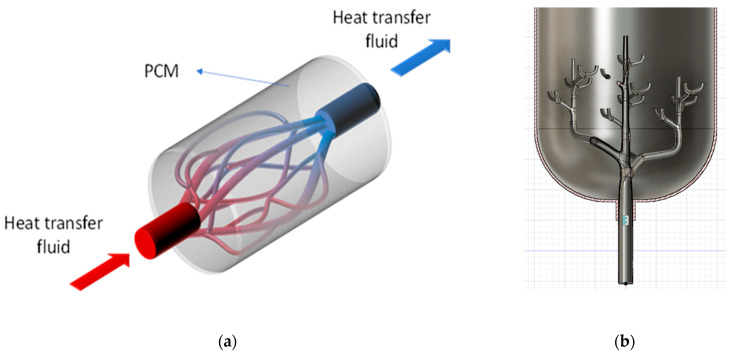
Schematic diagram showing the (**a**) bio-inspired TES tank concept and (**b**) arrangement of tubes in a shell. Adapted from Cabeza et al. (2024) [11].

The bio-inspired TES tank design investigated in this study was based on a bio-mimetic branching structure, inspired by natural vascular networks found in trees and leaves, as shown in Figure 1a, demonstrating the bio-inspired design concept, which mimicked vascular structures found in nature, such as plant stems or blood vessels. This hierarchical branching design maximized the surface area available for heat ex-change while ensuring an efficient distribution of working fluids throughout the shell. The design consisted of a main inlet pipe (parent) that branched into multiple smaller pipes (children) at different levels, creating a hierarchical structure that maximized heat transfer surface area while maintaining efficient fluid distribution, as shown in Figure 1b. This arrangement of tubes in Figure 1b showed the systematic reduction in pipe diameters and angles, which ensured optimal flow paths and minimized thermal resistance.

Advancing prior research in biomimetic TES tanks, this study focused on optimizing a shell-and-tube TES tank configuration with an internal branching network. The design drew inspiration from the hierarchical structure of natural vascular systems, particularly the efficient fluid distribution patterns found in leaf venation and tree branching systems. The internal pipe geometry followed a recursively branching dendritic structure, where pipe diameters tapered at each branching level, similar to the natural narrowing of vessels in biological systems. This fractal-like arrangement aimed to maximize the heat exchange surface area between the pipe walls and the surrounding phase change material while ensuring efficient fluid distribution throughout the system.

The integration of biomimetic principles with practical bio-inspired TES tank design considerations resulted in a structured approach to thermal energy storage. The design incorporated multiple levels of branching, each serving a specific role in heat transfer and fluid distribution. This hierarchical arrangement, combined with the careful consideration of geometric parameters at each level, created the foundation for the systematic optimization of the TES tank design. The following section detailed the specific design parameters that were identified as critical for optimizing the thermal performance of this bio-inspired system.

### 3.2. Design Parameters

**Figure 2:**
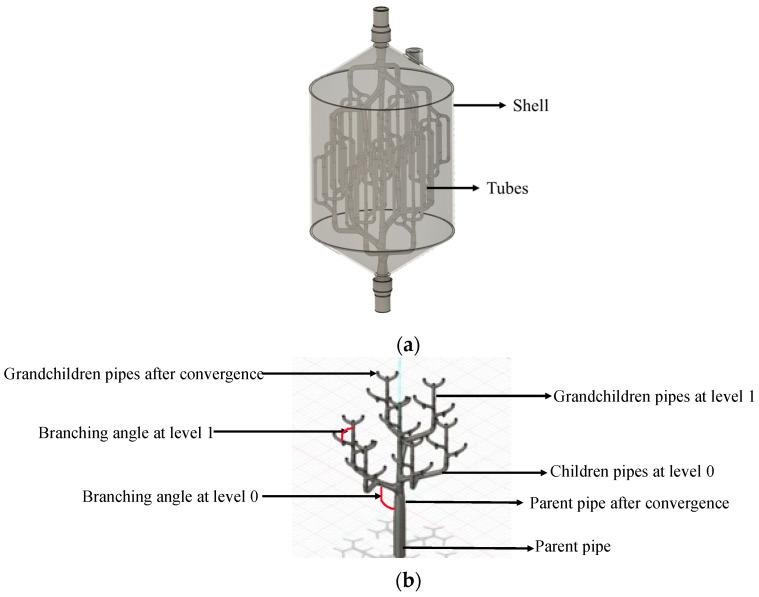
(**a**) Three-dimensional representation of the bio-inspired TES tank and (**b**) hierarchical branching structure. Adapted from Mehraj et al. (2024) [65].

The optimization framework addressed thirteen critical geometric parameters that defined the hierarchical heat transfer network, each parameter carefully bounded to ensure both performance enhancement and manufacturing feasibility. Figure 2 illustrates this hierarchical structure, where (a) showed the three-dimensional representation of the bio-inspired TES tank and (b) highlighted the complete geometry of the branching network that influenced the TES tanks performance. The selection of these parameters was driven by their direct impact on heat transfer surface area and fluid distribution efficiency.

Figure 2 presents a 3D representation of the complete tank, showcasing its overall configuration and the integration of branching networks within the cylindrical shell. It also provided a detailed view of the progressive branching geometry, emphasizing the transition from the parent pipe to smaller branches. This hierarchical design ensured a balanced trade-off between structural integrity and thermal efficiency. The systematic reduction in pipe diameters and optimization of branching angles promotes a uniform distribution of thermal loads while minimizing pressure losses. These design features were critical in enhancing heat transfer performance and maintaining operational reliability. The figure underscored the synergy between biomimetic principles and engineering innovation, offering a blueprint for optimized thermal energy storage systems.

The foundation of the design began with the parent pipe configuration, which established the primary flow path through the system. The parent pipe length determined the vertical reach of the heat transfer network, while its initial diameter influenced the overall flow capacity. A critical feature of the parent pipe was its gradual tapering, represented by the final diameter parameter, which facilitated the smooth transition into the primary branches and helped in maintaining uniform flow distribution throughout the network.

The branching structure consisted of two hierarchical levels, each playing a distinct role in thermal performance. At the first level (level 0), the design incorporated primary branches emerging from the parent pipe, characterized by their number, length, diameter, and branching angle. These parameters governed the primary distribution of heat transfer fluid and established the basic spatial coverage of the heat exchanger. The secondary level (level 1) created a more refined distribution network through additional branches sprouting from the primary branches. This dual-level hierarchy created a dense network that maximized contact with the phase change material while maintaining efficient fluid flow throughout the system.

The interaction between these geometric parameters created an integrated system where modifications to any single parameter could significantly impact overall performance. The parent pipe characteristics influenced the flow patterns through the entire network, while the branching parameters at each level determined both the spatial distribution of heat transfer surfaces and the flow resistance through the system. This complex interplay of parameters made the design optimization particularly suitable for genetic algorithm implementation, where each parameter represented a gene in the chromosome. This approach enabled a systematic exploration of different configurations while considering both performance optimization and manufacturing constraints.

Table 3 presents the dimensions of the original bio-inspired TES tank (Figure 2) design that was experimentally validated by Cabeza et al. (2024) [11]. These dimensions served as the baseline for our optimization study. The dimensions of the tubes depicted in Figure 2a represented one-half of the TES tank tube structure. The other half was a mirrored replica of this design, and, when joined together, they formed the complete structure of the heat exchanger tubes.

### 3.3. Genetic Algorithm: Principles and Implementation for TES Tank Design

Genetic algorithms (GAs) emerged as a computational method that mimicked biological evolution to solve complex optimization problems. The inherent ability of GAs to handle multiple simultaneous parameters while avoiding local optima made it particularly suitable for TES tank design optimization, where numerous geometric parameters affected thermal performance.

The fundamental GA process consisted of several key steps, best illustrated through a simple example. For the optimizing diameter of a single pipe for maximum heat transfer, the algorithm began by creating multiple random designs, forming an initial population with different diameters. The performance of each design was evaluated through a fitness function measuring the heat transfer rate. Better-performing designs were selected for reproduction, where their characteristics merged through crossover operations to create new designs. Occasional random modifications, known as mutations, prevented the solutions from becoming trapped in local optima. Through successive generations, the population evolved toward optimal pipe dimensions, demonstrating how genetic algorithms naturally progress toward improved solutions while maintaining design diversity.

This basic principle extended to the more complex bio-inspired TES tank optimization problem that was detailed in the following section. The implementation utilized an evolutionary computation framework, handling multiple design parameters simultaneously. Each potential TES tank design was encoded as a chromosome, where individual genes represented specific geometric parameters such as pipe diameters, branching angles, and lengths. An adequately sized population provided sufficient diversity for effective optimization while maintaining computational efficiency.

The selection process employed a roulette wheel approach, where designs with larger heat transfer surface areas gained higher probabilities of selection for reproduction. The process mimicked natural selection, allowing superior designs to contribute more frequently to the next generation while maintaining population diversity. Crossover operations combined features from successful designs with a defined probability, creating offspring that inherited beneficial characteristics from both parents. Mutation operations introduced occasional random variations in the design parameters, helping maintain genetic diversity and preventing premature convergence to suboptimal solutions. A comprehensive fitness function evaluated each design based on its total heat transfer surface area, while a penalty system enforced manufacturing constraints by reducing the fitness scores of designs that violated practical limitations. This balanced approach ensured the evolution progressed toward both optimal performance and manufacturability.

The optimization process continued through multiple generations, each iteration building upon previous improvements while adhering to practical constraints. Early generations showed rapid improvements in design performance, followed by more gradual refinement as the population converged toward optimal solutions. This methodical approach transformed the complex task of optimizing multiple interdependent design parameters into a manageable process. The GAs evolutionary nature allowed it to explore diverse design possibilities while systematically refining successful features, ultimately yielding a TES tank design that balanced optimal performance with manufacturing feasibility.

### 3.4. Problem Implementation

#### 3.4.1. Problem Definition and Objectives

The optimization problem focused on enhancing the bio-inspired TES tank prototype shown in Figure 2. through a systematic application of GA. The implementation utilized Python version 3.12.3 with the DEAP (distributed evolutionary algorithms in Python) framework, building upon the fundamental branching design principles established in this study. The detailed algorithmic steps used in the optimization process are provided in Appendix A.

The objective function was explicitly defined as maximizing the heat transfer surface area while maintaining practical manufacturing constraints within the shell dimensions. This study employed a single-objective optimization framework, where manufacturing constraints, such as the physical dimensions of shell and manufacturability limits, were integrated into the objective function using a penalty mechanism rather than being treated as separate objectives. These constraints ensured a practical and feasible optimization process.

The optimization bounds were carefully derived from the manufacturing feasibility demonstrated in Cabeza et al. (2024) [11] experimental work. The shell dimensions (150 mm diameter and 155 mm height) were maintained as primary constraints, while the ranges for branching parameters were established based on successfully manufactured prototypes. The minimum pipe diameters (6 mm) and maximum branching angles (70°) were set according to the manufacturing capabilities demonstrated using additive manufacturing techniques. These constraints ensure that the optimization process explores only the design space proven to be physically realizable, maintaining practical manufacturability while seeking performance improvements.

The design space encompassed thirteen interconnected geometric parameters that defined the complete TES tank architecture. These parameters included the parent pipe characteristics, such as its length, initial diameter, and final diameter after tapering. The level 0 branching structure was characterized by branch length, diameter, quantity, and branching angle relative to the parent pipe. Similarly, level 1 branching incorporated parameters for length, diameter, quantity, and branching angle, creating a hierarchical structure that mimicked natural vascular systems. The tapering ratios at each level ensured smooth flow transitions while maximizing heat transfer surface area.

The genetic encoding of these parameters followed a systematic nomenclature:

(a) Parent length—length of the main inlet pipe (mm);
(b) Parent diameter—outer diameter of the main inlet pipe (mm);
(c) Final parent diameter—final outer diameter of the main inlet pipe after tapering (mm);
(d) Children pipe length—length of branches after first bifurcation (mm);
(e) Children pipe diameter—outer diameter of branches after first bifurcation (mm);
(f) Final diameter of children pipe—final outer diameter of branches after tapering (mm);
(g) Grandchildren pipe length—length of branches after second bifurcation (mm);
(h) Grandchildren pipe diameter—outer diameter of branches after second bifurcation (mm);
(i) Final diameter of grandchildren pipe—final outer diameter of branches after tapering (mm);
(j) Number of branches at level 0—number of branches at the first bifurcation level;
(k) Branching angle at level 0—branching angle at the first bifurcation level (degrees);
(l) Number of branches at level 1—number of branches at the second bifurcation level;
(m) Branching angle at level 1—branching angle at the second bifurcation level (degrees).

Table 4 presents the optimization bounds for each parameter, defining the search space explored by the GA. These bounds were selected to balance thermal performance enhancement with practical manufacturability, ensuring that all generated configurations adhered to real-world fabrication constraints.

#### 3.4.2. Implementation of Architecture

The GA implementation was configured with carefully tuned control parameters to ensure efficient exploration of the TES tank design space while maintaining manufacturability constraints. Table 5 presents the key parameters governing the evolutionary process, which were optimized through preliminary testing. The population size of 100 provided a balance between genetic diversity and computational efficiency. A crossover probability of 0.3 was used to limit excessive recombination, ensuring the preservation of beneficial design traits while still allowing exploration of alternative configurations. The mutation probability of 0.7 was set to introduce variation in the branching parameters, preventing premature convergence and ensuring adequate search of the design space.

The tournament selection method, with a tournament size of 3, was used to manage selection pressure. This approach ensured that individuals with higher fitness values were more likely to be selected for reproduction while maintaining population diversity. The selection pressure provided a balance between intensifying the search for near-promising solutions and maintaining genetic diversity to explore the broader design space effectively.

An individual probability distribution of 0.5 governed the likelihood of specific parameters being selected for modification during mutation operations. The distribution index value of 39 controlled offspring similarity to parents during crossover and mutation operations. Higher distribution index values generated offspring more like parents, while lower values enable more diverse solutions. The optimization process continued through 500 generations, providing sufficient iterations to capture both rapid early improvements and subsequent design refinements. The control parameters for the GA implementation were tuned through preliminary testing, with bounds informed by the physical constraints of the original experimental design [11]. This approach ensured that the optimization process respected both theoretical performance objectives and practical manufacturing limitations demonstrated in previous experimental validation.

#### 3.4.3. Optimization Process

**Figure 3:**
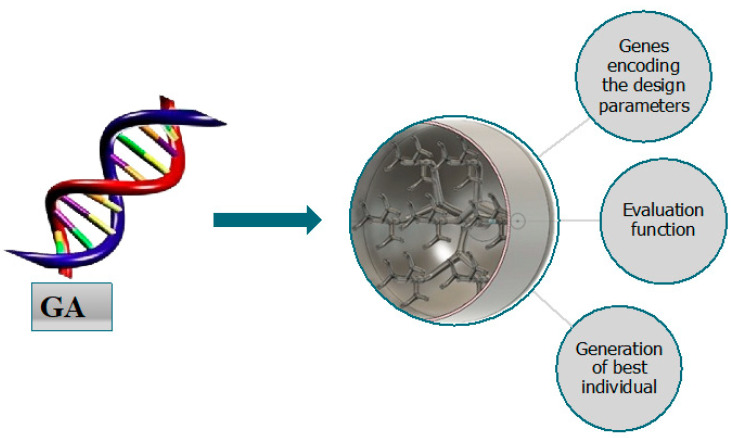
AI optimization of TES tank design using GA.

The optimization process implemented a systematic evolutionary approach using the carefully selected control parameters shown in Figure 3 and Table 6. These parameters were chosen to balance exploration of the design space with computational efficiency while maintaining population diversity. Figure 3 outlined the GA-based optimization process. The algorithm began with the random initialization of the population, followed by a fitness evaluation based on the exposed surface area of the design. Through iterative applications of selection, crossover, and mutation, the population evolved toward optimal solutions. Critical parameters, such as population size, mutation probability (0.7), and crossover probability (0.3), governed the efficiency and diversity of the optimization process. The iterative loop continues for 500 generations to ensure a thorough exploration of the design space.

The process began with a population initialization through a random generation of feasible designs, with each design undergoing rigorous constraint verification before entering the population pool. Initial fitness evaluations established baseline performance metrics, creating the foundation for evolutionary improvements. The population size of 100 individuals provided sufficient genetic diversity while keeping computational costs manageable.

The selection mechanism utilized two complementary methods. The primary method employed a roulette wheel approach, where selection probability correlated directly with fitness values. This was augmented with tournament selection (size 3) for parent identification, ensuring superior designs had higher chances of contributing genetic material to subsequent generations. This dual-selection approach helped maintain population diversity through the careful balancing of exploitation and exploration tendencies.

Genetic operators were implemented with adaptive probabilities, as shown in Table 4. One-point crossover operations, operating at rates between 0.3 and 0.7, combined beneficial features from parent designs. Mutation operations, using bit-flip mutation at rates of 0.1–0.3, introduced controlled variability to prevent premature convergence. The evolution control system monitored both generation count and population diversity, performing convergence checks every 10 generations to maintain effective search dynamics throughout the optimization process.

The one-point crossover and bit-flip mutation operators were chosen for their simplicity and proven effectiveness in high-dimensional optimization problems. These standard operators facilitated a systematic exploration of the complex design space while maintaining computational efficiency, aligning with the objective of this study, which is to obtain manufacturable and scalable solutions.

**Figure 4:**
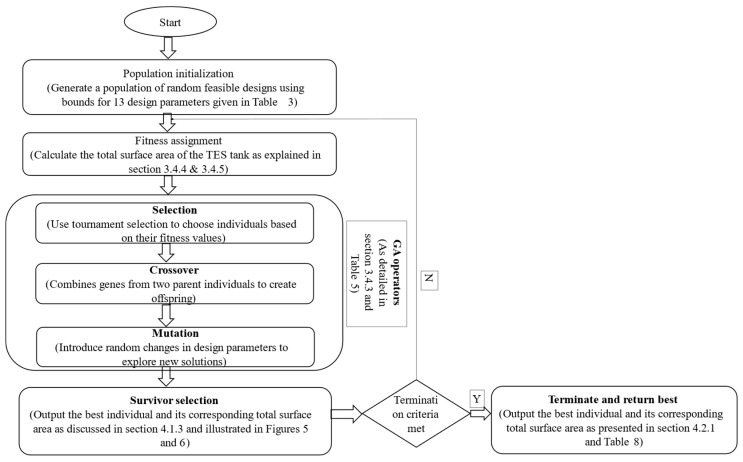
Optimization process workflow.

The optimization workflow illustrated in Figure 4 highlights the iterative nature of the genetic algorithm (GA). The process began with the initialization of the population, followed by the fitness evaluation, and progressed through the genetic operations of selection, crossover, and mutation. The green feedback loop represented the evolutionary progression, where each generation builds upon improvements from the previous ones. This iterative process refined the design parameters, gradually converging toward a high-performance TES tank configuration. The feedback loop between fitness evaluation and genetic operations ensured a balance between exploring new designs and exploiting high-performing solutions. Convergence was achieved after 500 generations, as defined by the termination criterion, at which point the optimal solution is returned.

#### 3.4.4. Fitness Function Implementation

The optimization process employed a fitness function that evaluated the heat transfer potential of each candidate design based on the total heat transfer surface area of the TES tank. This hierarchical calculation mirrored the bio-inspired branching structure of the design and formed the core objective function for the optimization. The fitness function, defined by the total heat transfer surface area $A_{T}$, served as the objective function for the optimization process. Constraints related to shell dimensions, branching angles, and pipe diameters were enforced through the feasibility function and penalty mechanism. These mathematical formulations collectively ensure that the optimization remained focused on manufacturable, high-performance designs [66,67].

The total exposed surface area of the bio-inspired TES tank was calculated in Equation (1) as [68] follows:

(1) $$A_{T}=2*\left(A_{P}+A_{C}+A_{Gc}\right)$$

where $A_{p}=pi*\left(P_{r1}+P_{r2}\right)*s_{P}$ was the surface area of the parent channel, $A_{C}=n_{b0}*pi*\left(C_{r1}+C_{r2}\right)*s_{C}*cos\left(angle_{level\;0}\right)$ was the surface area of level 0 branches, and $A_{Gc}=n_{b1}*pi*\left(Gc_{r1}+Gc_{r2}\right)*s_{Gc}*cos\left(angle_{level1}\right)$ was the surface area of level 1 branches.

Here, $P_{r1}$ and $P_{r2}$ were the initial and final radii of the parent channel, $C_{r1}$ and $C_{r2}$ represent the initial and final radii of the level 0 branches, and $GC_{r1}$ and $GC_{r2}$ corresponded to the level 1 branches. The slant heights $S_{P}$, $S_{C}$, and $S_{Gc}$ for the parent channel, level 0, and level 1 branches, respectively, were calculated using the Pythagorean theorem as [69] follows:

$s_{P}=\sqrt{{\left(P_{r1}-P_{r2}\right)}^{2}+{\left(P_{length}\right)}^{2}}$, $s_{C}=\sqrt{{\left(C_{r1}-C_{r2}\right)}^{2}+{\left(C_{length}\right)}^{2}}$, $s_{Gc}\sqrt{{\left(Gc_{r1}-Gc_{r2}\right)}^{2}+{\left(Gc_{length}\right)}^{2})}$.

The angles $\left(angle_{level\;0}\right)$ and $\left(angle_{level\;1}\right)$ represented the branching angles for level 0 and level 1 branches, respectively. The $n_{b0}$ and $n_{b1}$ denoted the number of branches at level 0 and level 1.

This fitness function enabled the GA to evaluate candidate designs systematically and drove the optimization toward configurations that maximize surface area while adhering to manufacturing constraints. The multiplication by two reflected the symmetrical nature of the design, where the outlet section mirrors the inlet configuration.

#### 3.4.5. Constraint Checking Function

The feasibility of each candidate solution was evaluated using a constraint-checking function. This function ensured that the design remained within predefined dimensional and manufacturing limits. A candidate solution was considered feasible if it satisfied the following constraints:

a. Dimensional constraints:
  - The total vertical length of the design must not exceed the shell height:
    $$\left(P_{length}+C_{length}\right)\leq 155\;\mathrm{mm}$$
  - The maximum diameter must not exceed the shell diameter:
    $$max\left(P_{d}+C_{d}\right)\leq 150\;\mathrm{mm}$$
    where $P_{length}$, $C_{length}$, $P_{d}$, and $C_{d}$ represented parent pipe length, children pipe length, parent pipe diameter, and children pipe diameter, respectively.
b. Geometric constraints:
  - The branching angles must remain within feasible limits:
    $$30{}^{\circ}\;\leq \;(angle_{level\;0}),\;(angle_{level\;1})\leq 70{}^{\circ}$$
  - The pipe diameters must stay within manufacturing tolerances:
    $$6\;\mathrm{mm}\;\leq \;C_{d},\;GC_{d}\leq 10\;\mathrm{mm}$$

Here $\left(angle_{level\;0}\right)$, $\left(angle_{level\;1}\right)$, $C_{d}$, and G$C_{d}$ represented the branching angles for level 0 and level 1 branches, as well as the diameters of the children and grandchildren pipes, respectively.

The feasibility function was implemented as follows:

$$\;feasible\left(individual\right):\;\;if\left(P_{length}+C_{length}\right)>SHELL_{LENGTH}\;\;returnFalse\;\;ifmax\left(P_{d}+C_{d}\right)>SHELL_{DIAMETER}\;\;returnFalse\;returnTrue$$

To handle constraint violations, a penalty-based approach was employed within the fitness function. The penalized fitness function was defined in Equation (2) as [70] follows:

(2) $$f\left(x\right)=\left\{\begin{array}{c} \;f\left(x\right),\;\;if\;all\;the\;constraints\;are\;satisfied,\\ f\left(x\right)-P\cdot \overset{n}{\underset{i=1}{\sum }}V_{i},\;\;if\;constraints\;are\;violated \end{array}\right.$$

where f(x) represented the fitness value, P was a penalty multiplier, and $V_{i}$ quantifies the degree of violation for each constraint i. This ensured infeasible solutions were assigned lower fitness values, steering the optimization toward compliant designs.

For designs that violated any constraints, a penalty was applied during fitness evaluation:

$$F_{Penalized}=F_{Unpenalized}-P_{Violation},$$

where $P_{Violation}$ was proportional to the degree of constraint violation. This ensured that infeasible solutions were assigned lower fitness values, steering the optimization process toward compliant designs.

This constraint-handling mechanism guided the optimization algorithm to prioritize feasible designs while maintaining focus on maximizing the total heat transfer surface area. The penalty-based approach ensured that infeasible solutions were assigned to lower fitness values, steering the genetic algorithm toward compliant and high-performing designs.

#### 3.4.6. Validation Framework

The validation framework implemented a three-tiered approach focusing on computational verification of the GA optimization process, as detailed in Table 7. This structure ensured solution feasibility and convergence quality throughout the evolutionary process.

The validation framework served multiple purposes throughout the optimization process, with each tier addressing specific aspects of design verification. At the geometric level, computational validation focused on ensuring physical feasibility by verifying that all designs remained within the prescribed shell dimensions of a 150 mm diameter and 155 mm height. This included rigorous checks of minimum tube spacing requirements and a validation of branching angles to maintain manufacturing feasibility. The performance assessment constituted the second validation tier, where design effectiveness was evaluated through detailed surface area calculations and systematic comparison with the baseline bio-inspired TES tank design from the literature. This assessment focused specifically on quantifying surface area improvements while ensuring adherence to manufacturing constraints of the original design. The final validation tier involved convergence analysis, which monitored optimization progress through systematic tracking of fitness values and population diversity across generations. This comprehensive monitoring revealed that improvements plateaued after approximately 250 generations, providing a clear termination point for the optimization process. Through this multi-tiered approach, the validation framework successfully ensured that the optimization process generated feasible designs with enhanced surface area characteristics while maintaining the practical manufacturing constraints established by the original bio-inspired TES tank design.

To ensure the robustness and consistency of the optimization results, the GA was executed 30 times with varying random seeds. This approach aimed at evaluating the repeatability of the optimization process under different initial conditions. For each run, key statistical measures, including the mean fitness value, standard deviation, and confidence intervals, were calculated to assess the reliability and convergence behavior of the GA. These metrics provided a comprehensive understanding of the performance of the algorithms across multiple independent runs.

#### 3.4.7. Parameter Sensitivity Analysis Framework

To assess the influence of GA parameters on optimization performance, a sensitivity analysis was conducted. The parameters analyzed included crossover probability (CxPb), mutation probability (MutPb), population size, and the number of generations. Each parameter varied systematically within reasonable bounds (e.g., ±10%, ±20), while other parameters were held constant at their baseline values (CxPb = 0.3, MutPb = 0.7, population size = 100, generations = 500). For each variation, the GA was executed, and the resulting fitness value (F) was recorded.

The sensitivity index (S) for each parameter was calculated as [71] follows:

$$S=\frac{\Delta F}{F_{baseline}}$$

where ΔF represents the change in the fitness value and F_baseline_ denoted the fitness value for the baseline parameter set. This iterative process enabled quantification of the relative impact of each parameter on the optimization performance.

The results were visualized by plotting normalized sensitivity indices against the percentage perturbations for each parameter. This analysis assumed that the fitness function variance arises solely from individual parameter changes, ignoring interactions between parameters. The findings provided valuable insights into the robustness of the GA implementation and highlighted parameters that required tighter control to ensure optimization stability and efficiency.

## 4. Results and Discussions

### 4.1. Optimization Performance Analysis

#### 4.1.1. Geometric Parameter Optimization

The GA optimization process explored thirteen key geometric parameters within predefined ranges, guided by manufacturing constraints and biological design principles. Table 8 presents these parameters and their optimized values, representing the culmination of the evolutionary search process. The parameter evolution revealed distinct patterns in the optimization trajectory. The parent pipe parameters consistently evolved toward their upper bounds, with lengths reaching 45 mm and diameters reaching 13 mm, indicating a strong optimization preference for maximized primary flow capacity. In the branching structure, the algorithm identified optimal angles of 50° at both levels, balancing spatial coverage with flow efficiency. Branch quantities evolved differently at each level, with modest increases at level 0 but substantial expansion at level 1, suggesting a hierarchical optimization of the distribution network. The diameter reduction sequence (13 mm → 8 mm → 6 mm) emerged naturally through the optimization process, mirroring biological vessel tapering patterns.

**Figure 5:**
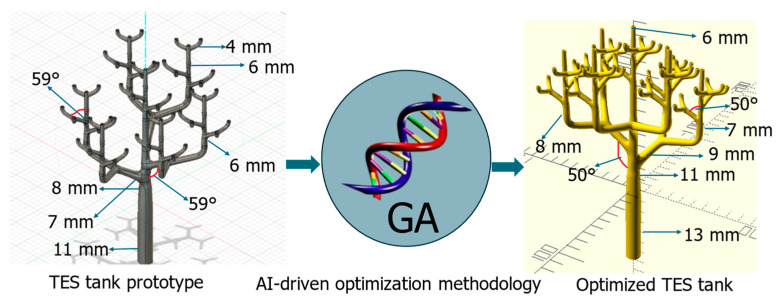
AI-driven geometric optimization of a bio-inspired TES tank using genetic algorithms.

Figure 5 illustrates the optimization process, showing the iterative evolution of the TES tank design under the GA. The left image represented the initial design configuration, while the right image demonstrated the final optimized structure. The genetic algorithm refined the branching structure while preserving manufacturability and fluid flow feasibility. This visualization highlighted how AI-driven optimization progressively improved the TES tank geometry by balancing biological efficiency with engineering constraints.

#### 4.1.2. Surface Area Enhancement

The optimization strategy focused on three primary mechanisms for surface area improvement. First, the spatial utilization strategy optimized branching angles and distributions to maximize coverage within the constrained shell volume. Second, the contact surface strategy refined branch quantities and positions to enhance heat transfer interface area. Third, the diameter optimization strategy balanced flow capacity with surface area through strategic tapering ratios. These strategies worked synergistically within the GA framework, each contributing to overall performance enhancement while maintaining practical constraints. The resulting design represents an optimal balance between these competing optimization objectives, demonstrating the GAs ability to navigate complex design trade-offs.

The 29% increase in surface area achieved through GA optimization translated directly into enhanced heat transfer capacity. This improvement was critical for TES tanks operating in industrial and renewable energy systems, where maximizing thermal exchange efficiency was vital for achieving faster charging/discharging rates and reducing thermal losses.

#### 4.1.3. Design Evolution

**Figure 6:**
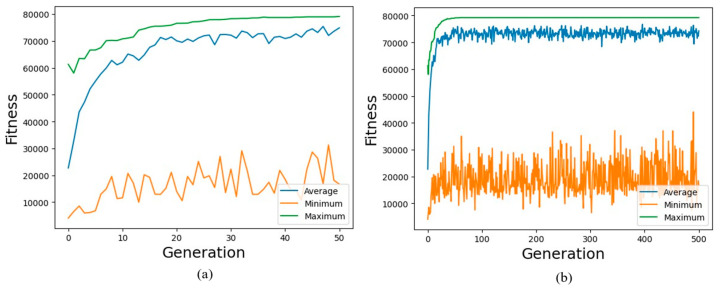
Evolution of individual fitness across generations during genetic algorithm optimization, showing average, minimum and maximum fitness values (**a**) Population size of 50 for 50 generations and (**b**) Population size of 500 for 500 generations.

The evolutionary progression of the optimization process demonstrated distinct phases of improvement, as illustrated in Figure 6. The fitness curve revealed three characteristic stages of evolution: rapid initial improvement, steady progression, and final convergence. During the first 50 generations, the surface area increased dramatically from 61,303.9 mm^2^ to 79,148.3 mm^2^, indicating efficient exploration of the design space. This rapid improvement phase reflected the ability of the algorithm to quickly identify and combine beneficial design features.

Figure 6a, in particular, highlighted a “hockey stick” pattern in the fitness curve during the early generations. This distinctive shape was characterized by an abrupt rise in the fitness value, followed by a gradual leveling off as the algorithm transitions from exploration to exploitation. The steep upward trend in the initial phase signifies the capability of the algorithm to rapidly capture high-impact improvements, such as refining branching angles and maximizing pipe diameters. This was analogous to the blade of the hockey stick, where significant progress is made. The subsequent tapering off, resembling the shaft of the stick, represented a shift towards incremental refinements in fitness as the population converges towards an optimal solution. This behavior underscored the effectiveness of the genetic algorithm in achieving rapid gains while maintaining steady progress toward convergence.

During the rapid improvement phase, the GA successfully identified critical design features, such as maximizing parent pipe diameter and refining branching angles, which significantly increased the heat transfer surface area. The subsequent refinement phase focused on fine-tuning these parameters, balancing spatial coverage and manufacturability. This gradual progression ensured that the design evolved towards a well-rounded configuration with optimized geometric features.

The intermediate phase, spanning generations 50–200, showed more gradual improvements as the algorithm refined the branching structure. The fitness value increased marginally from 79,148.3 mm^2^ to 79,200 mm^2^, suggesting a fine-tuning of parameters rather than major structural changes. This behavior aligns with the optimization objective of finding a balanced configuration that maximizes heat transfer surface area while maintaining practical constraints.

The convergence phase, observed beyond generation 200, exhibited asymptotic behavior with the fitness value stabilizing around 79,223.7 mm^2^. This plateau indicated the algorithm reached a near-optimal solution within the defined constraint boundaries. The termination at 500 generations was justified by the negligible improvement (less than 0.1%) in fitness values beyond generation 250, demonstrating efficient computational resource utilization. The stability of the fitness curve during the final generations reflects the ability of algorithm to balance exploration and exploitation effectively, ensuring robust convergence to high-quality solutions.

### 4.2. Comparative Analysis

#### 4.2.1. Parameter Comparison with Original Design

A systematic comparison between the GA-optimized design and the original bio-inspired TES tank prototype revealed significant adaptations across key geometric parameters, as shown in Table 9. The optimization process yielded strategic modifications in both the primary flow channels and branching architecture. In the primary channel structure, the inlet pipe diameter increased from 11 mm to 13 mm, accompanied by a corresponding increase in tapering diameters at level 0 (8 mm to 11 mm) and level 1 (6 mm to 7 mm). The branching configuration underwent substantial refinement, with both level 0 and level 1 branching angles reducing from 59° to 50°, optimizing spatial distribution efficiency. Notably, the number of branches increased at both levels, with level 0 branches expanding from 7 to 8 and level 1 branches from 49 to 64, enhancing the overall heat transfer network density. These coordinated modifications resulted in a significant enhancement of the total heat transfer area from 6.6 × 10^4^ mm^2^ to 7.9 × 10^4^ mm^2^, representing a 29% improvement while maintaining the original shell dimensions of a 150 mm diameter and 155 mm height. The moderate parameter deviations, typically ranging from 5 to 10%, suggested that the optimization process successfully identified improvements while remaining within practical manufacturing constraints, validating the GAs effectiveness in refining the bio-inspired design concept.

To further contextualize the significance of the GA-optimized design, a comparison with key studies from the literature was conducted, highlighting advancements achieved through biomimetic principles and optimization techniques. Table 10 summarized these comparisons, showcasing how the proposed method bridges the gap between prior approaches and practical implementation.

Compared to previous studies, the proposed GA-driven optimization uniquely balances practical manufacturability with biomimetic design principles, achieving a 29% improvement in surface area without requiring experimental revalidation or material modifications. For instance, Zhang et al. (2023) [39] achieved a 40–60% improvement in heat transfer using biomimetic oval geometries, but their designs lacked optimization and had limited generalization. Similarly, Huang et al. (2023) [41] focused on application-specific biomimetic fin structures for waste heat recovery but faced scalability challenges. Mohtasim and Das (2024) [40] demonstrated improved thermal conductivity through material-based enhancements but did not consider geometric or structural optimization. This study bridged these gaps by offering a systematic, geometric-driven optimization approach that was versatile and scalable for broader applications in thermal energy systems.

#### 4.2.2. Performance Implications

The geometric modifications generated in this study demonstrated several critical performance implications for bio-inspired TES tank designs. Building upon the experimental findings of Cabeza et al. (2024) [11], which reported a 52% faster discharge rate for the original bio-inspired design, the AI-generated configurations produced in this study achieved a 29% increase in heat transfer surface area through coordinated parameter refinements. This improvement suggested that further performance enhancements could be achieved without altering the fundamental manufacturing constraints of the original bio-inspired model.

The AI-driven design generation leveraged multiple bio-inspired geometric refinements that contributed to enhanced heat transfer efficiency. The primary channel diameters increased from 11 mm to 13 mm, improving fluid flow capacity while maintaining pressure drop stability. The branching angles were modified from 59° to 50°, optimizing spatial heat distribution and ensuring better thermal interaction between the heat transfer fluid (HTF) and the phase change material (PCM). Additionally, a 30% increase in branch density at level 1 led to a more extensive contact area between HTF and PCM, further improving the efficiency of heat exchange.

These modifications aligned with previous studies on biomimetic heat exchanger optimization, where hierarchical branching networks demonstrated up to 40–60% improvement in thermal distribution efficiency (Zhang et al., 2023; Huang et al., 2023) [39,41]. However, unlike prior works that primarily explored biomimetic geometries in a theoretical framework, this study implemented a computational methodology that systematically generated manufacturable TES tank designs. This distinction ensured that the proposed configurations were not only thermally efficient but also practical for real-world implementation.

Beyond heat transfer enhancement, the manufacturability and feasibility of the generated designs remained key considerations in TES tank applications. The designs deviated only 5–10% from the original prototype, suggesting a straightforward adaptation to existing manufacturing processes. The preservation of symmetrical branching patterns and the consistent angle distributions facilitated compatibility with additive manufacturing techniques, ensuring structural integrity while maintaining practical scalability. The optimized configurations retained critical bio-inspired structural features while improving the heat transfer surface area, making them suitable for real-world applications.

The results of this study indicated that AI-driven biomimetic design approaches could be effectively applied to TES tank engineering, bridging the gap between theoretical design enhancements and real-world implementation. While previous research demonstrated the benefits of biomimetic designs in thermal management, this study further reinforced the applicability of AI-generated designs for large-scale manufacturing. Future work should focus on experimental validation of these computationally generated TES tank models to confirm their thermal performance gains and industrial feasibility. Additionally, further studies should explore the integration of material-based enhancements, such as high-conductivity PCM composites, to maximize energy storage efficiency in practical settings.

### 4.3. Design Validation

#### 4.3.1. Constraint Satisfaction

The GA-optimized design successfully satisfied all imposed manufacturing constraints while achieving performance improvements. The primary dimensional constraints of a 150 mm shell diameter and 155 mm shell height were strictly maintained throughout the optimization process. The total vertical reach of the final design, combining parent pipe length (45 mm) and branch lengths (30 mm at level 0), remains within the shell height limitation. Similarly, the maximum radial spread, determined by branching angles and lengths, stays within the shell diameter constraint. The optimized branching angles of 50° at both levels, reduced from the original 59°, contributed to more efficient space utilization while maintaining structural integrity and manufacturability. The graduated reduction in pipe diameters from 13 mm at inlet to 7 mm at terminal branches aligned with standard manufacturing capabilities and ensured consistent flow distribution.

#### 4.3.2. Surface Area Calculations

The surface area validation revealed consistent improvement across multiple optimizations runs, confirming the reliability of the achieved enhancement. The total heat transfer surface area obtained from the optimized design reached 7.9 × 10^4^ mm^2^, verified through both analytical calculations and comparative geometric analysis. This represents a 29% improvement over the original design with a surface area of 6.6 × 10^4^ mm^2^, demonstrating consistent enhancement across all branching levels. The reliability of these calculations was validated through multiple methodologies, including direct geometric measurements and computational verification. The enhanced surface area directly correlates with the design objectives of improving thermal performance through increased heat transfer potential. The systematic validation process confirmed that the surface area improvements were achieved while maintaining uniform distribution across the branching network, suggesting potential for enhanced thermal performance in practical applications. This validation established a robust foundation for the practical implementation of the optimized design, confirming both its theoretical advantages and manufacturing feasibility.

### 4.4. Optimization Performance Assessment

**Figure 7:**
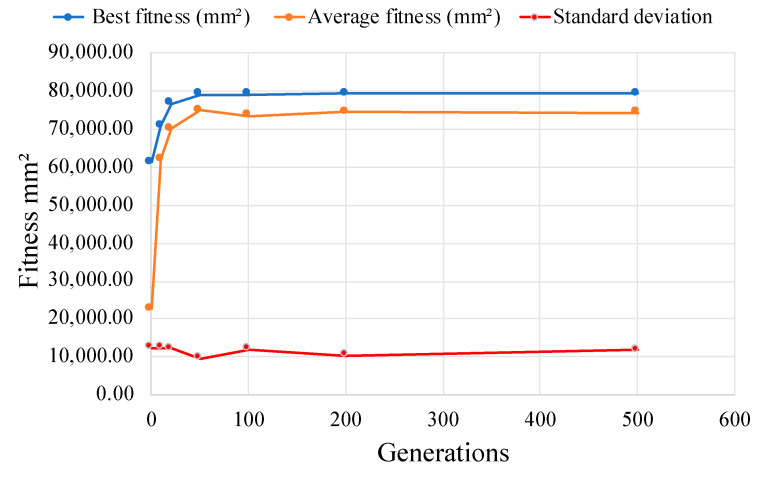
Comprehensive optimization performance analysis showing convergence profile data (fitness vs. generations).

The statistical analysis of the optimization process, as depicted in Figure 7, demonstrated distinct performance trends that validate the effectiveness of the algorithm. Figure 7 shows a clear reduction in fitness variance across generations, indicating population convergence. The gradual decline in standard deviation reflected the stabilization of the population as it nears the optimal design. This convergence profile confirmed the capability of GA to consistently achieve high-performing designs within the specified 500 generations.

The inclusion of diversity metrics further strengthened the analysis of convergence behavior. Figure 7 provides additional insights into the genetic diversity of population throughout the optimization process. During the initial exploration phase (generations 0–50), the population maintained high diversity, as indicated by a standard deviation of 12,378 mm^2^ and a coefficient of variation of 54.5%. This enabled the effective exploration of the design space and rapid improvement, with the fitness value increasing from 61,303.9 mm^2^ to 79,148.3 mm^2^, representing an average improvement rate of 356.9 mm^2^/generation, as clearly illustrated by the steep slope in the convergence plot.

The intermediate refinement phase (generations 50–200) showed more gradual improvements, with fitness values increasing marginally from 79,148.3 mm^2^ to 79,200 mm^2^. During this phase, the diversity metrics indicated a reduction in standard deviation to 10,405.80 mm^2^ and a coefficient of variation to 13.9%, as shown in Table 11. This reflected a balanced trade-off between exploration and exploitation.

The final convergence phase (generations 200–500) exhibited asymptotic behavior, with fitness values stabilizing at 79,223.7 mm^2^, as depicted by the nearly horizontal line in Figure 7. Diversity metrics during this phase revealed a standard deviation of approximately 11,777 mm^2^ and a coefficient of variation of 15.8%. These metrics confirm that the population maintained sufficient diversity to avoid premature stagnation while achieving convergence toward uniform, optimized solutions.

The diversity metrics provided evidence of the robustness of the algorithm in exploring the high-dimensional design space and ensuring convergence to high-quality solutions. The consistent reduction in the standard deviation and coefficient of variation over the optimization process validated the ability of algorithm to stabilize the population and achieve reliable, practical designs for TES implementations.

Comprehensive performance metrics, augmented with diversity analysis, demonstrate robust optimization behavior characterized by initial rapid improvement (shown by the steep slope in Figure 7), controlled refinement (moderate slope), and stable convergence (near-horizontal slope). These findings validated the effectiveness of the GA implementation, the chosen control parameters, and the reliability of the optimized design.

#### 4.4.1. Statistical Validation of Optimization Consistency

**Figure 8:**
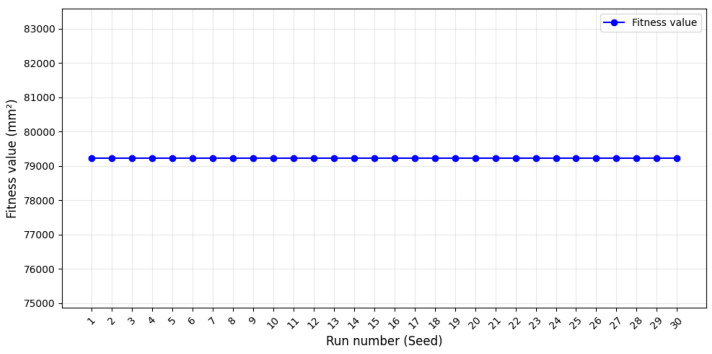
Statistical validation of optimization consistency.

To validate the reliability of the optimization process, statistical analysis was conducted on 30 independent optimization runs with varying random seeds. The results are visually represented in Figure 8, which illustrates the fitness values obtained across all runs. The graph highlighted the minimal fluctuation in fitness values, demonstrating consistent optimization performance. A mean fitness value of 79,223.7 mm^2^ was observed, with an exceptionally low standard deviation of 1.45 × 10^−11^ mm^2^, indicating minimal variation between runs. Additionally, the confidence interval for the fitness values was calculated to be precisely 79,223.7 mm^2^, highlighting the consistency of the GA.

This exceptionally narrow range demonstrates that all optimization runs converged to nearly identical solutions, providing strong evidence of the robustness of the algorithm and its reliability in achieving near-optimal results.

The confidence interval, standard deviation (s), and mean performance (μ) were calculated using the formula as described in the standard statistical literature (Montgomery et al. (2010)) [72]:

1. $Confidence\;interval=\left(\bar{x}-Z\cdot \frac{s}{\sqrt{n}},\overset{-}{x}+Z\cdot \frac{s}{\sqrt{n}}\right)$
  where $\overset{-}{x}$ = mean of the sample data, s = standard deviation of the sample data, n = number of samples, and Z: Z-score corresponding to the desired confidence level.
2. $s=\sqrt{\frac{\sum_{i=1}^{n}{\left(x-\bar{x}\right)}^{2}}{n}}$
  where s = standard deviation of the dataset, x = individual data points, $\bar{x}$ = mean of the dataset, n = of data points.
3. $\mu =\frac{1}{n}\overset{n}{\underset{i=1}{\sum }}x_{i}$
  where μ = mean performance, n = total number of independent runs (e.g., n = 30), and $x_{i}$ = fitness value obtained in the i^th^ run.

These findings demonstrated the stability of the algorithm and its effectiveness in exploring the high-dimensional design space of the TES tank. The consistent performance across multiple runs validated the suitability of GA for handling complex design challenges with stringent constraints. Moreover, the minimal variation between runs confirmed that the optimization process reliably avoids local optima, ensuring robustness and practical applicability of the AI-driven approach for TES tank development.

#### 4.4.2. Sensitivity Analysis of Geometric and Design Parameters

The robustness of the optimization process was evaluated through a parameter sensitivity analysis, which investigated the impact of variations in individual design parameters on the total heat transfer surface area. The parameters analyzed included the parent channel dimensions, branching angles, branch diameters, branch lengths, and the number of branches at levels 0 and 1. Each parameter was systematically varied within its predefined bounds, while all other parameters were held constant at their optimized values.

**Figure 9:**
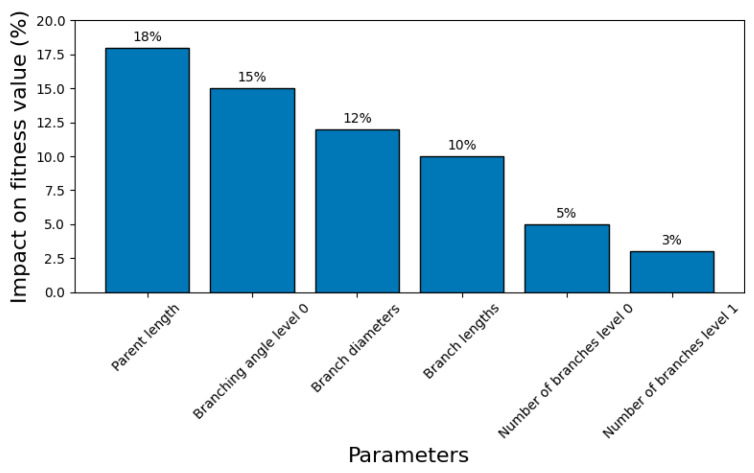
Parameter sensitivity analysis.

The results, as illustrated in Figure 9, reveal that geometric parameters, particularly the parent channel length and the branching angles at level 0, exhibited the highest sensitivity. A 10% variation in these parameters led to changes of up to 18% in the total fitness value. Branch diameters also showed a moderate influence, causing fitness value variations of approximately 12% for similar adjustments. In contrast, the number of branches at both levels demonstrated minimal sensitivity, with fitness value changes in less than 5%.

These findings highlighted the critical role of precise control over geometric parameters, particularly parent channel dimensions and branching angles, in achieving optimal thermal performance. The relatively low sensitivity of the number of branches underscored the robustness of the genetic algorithm in maintaining performance despite variations in this parameter.

The sensitivity analysis offered valuable insights into parameter prioritization, ensuring that design and manufacturing efforts focus on the most influential variables. The observed trends aligned with established principles in thermal energy storage, where branching angles and parent channel dimensions played a critical role in heat transfer efficiency. By identifying these key parameters, the analysis enhanced the overall reliability and practicality of the optimized TES design. These findings further validated the effectiveness of the GA-driven approach in optimizing TES structures for real-world applications.

### 4.5. Computational Cost Assessment and Comparison

The computational efficiency of the genetic algorithm (GA) implementation was evaluated by measuring the total time required for optimization and comparing it with alternative optimization methods reported in the literature. For the current GA setup (population size = 100, generations = 500), the total computational time was recorded as 3.51 s for simulations on a standard workstation with an Intel Core i7 processor.

A comparative analysis, as shown in Table 12, was conducted using reference computational studies on optimization tasks employing methods such as particle swarm optimization (PSO) and simulated annealing (SA). While the studies by Richa et al. (2024) [73] and Jillani et al. (2024) [74] do not focus specifically on thermal energy system (TES) optimization, they provided indicative computational costs for these methods under similar parameter scales, such as comparable population sizes and iteration numbers. These insights offered a general understanding of computational performance but should be interpreted with caution, given the potential differences in problem domains and setups.

This comparative analysis highlighted the GAs’ effectiveness not only in generating high-performing designs but also in optimizing computational resource utilization. Its computational efficiency significantly reduced design iteration time while enhancing scalability for large-scale TES applications. By achieving superior designs at a low computational cost, the GA proved to be a highly suitable approach for complex, high-dimensional optimization problems, particularly in rapid prototyping and industrial implementation.

## 5. Limitations and Future Research Directions

This study demonstrated the potential of an AI-driven biomimetic framework for TES tank design using genetic algorithms; however, several limitations warranted consideration and provided direction for future research.

One primary limitation stemmed from the stochastic nature of genetic algorithms, which, despite achieving significant improvements, did not guarantee absolute global optimality. The current implementation employed a real-valued vector representation, which may have constrained the exploration of more diverse branching geometries. Future work could explore alternative genetic representations or hybrid approaches to introduce greater design diversity while maintaining manufacturability.

The optimization framework focused on maximizing heat transfer surface area as a proof-of-concept. However, a more comprehensive fitness function could further enhance the thermal performance characteristics of the TES tank. Future optimization frameworks should integrate additional parameters, such as pressure drop minimization, storage capacity maximization, uniformity of pipe distribution, and material volume minimization for cost efficiency. Implementing multi-objective optimization would allow for a more balanced design approach, capturing both thermal and structural considerations.

While this study concentrated on geometric design generation, future research should expand the scope to include material properties and fluid characteristics. Incorporating parameters such as thermal conductivity, specific heat capacity, and fluid flow properties could significantly improve the accuracy of performance predictions. Additionally, computational fluid dynamics (CFD) simulations would provide a more detailed evaluation of heat transfer efficiency and pressure drop effects, supporting further refinement of TES designs.

A critical next step involves the physical implementation and experimental validation of the generated TES tank designs. While the current study produced designs closely resembling experimentally validated configurations, fabrication and real-world testing are necessary to confirm practical feasibility. Future work will focus on prototype fabrication, experimental testing, and comparative performance analysis against conventional TES designs. Since Cabeza et al. (2024) [11] successfully 3D-printed similar bio-inspired TES designs at lower costs than conventional shell-and-tube tanks, the cost-effectiveness of AI-generated designs can also be evaluated through real-world implementation.

The optimization framework itself presents several opportunities for enhancement. Future research could explore the following:

1. Hybrid optimization approaches that combine GA with local search methods for improved convergence;
2. Parallel computing strategies to enable larger population sizes and deeper design exploration;
3. Advanced fitness functions incorporating multiple performance metrics, including thermal efficiency, manufacturability, and material constraints;
4. Integration of machine learning techniques for adaptive optimization, allowing the algorithm to learn from previous design iterations and refine search strategies.

This study laid the foundation for AI-driven biomimetic TES tank designs, but long-term objectives include multi-objective optimization, dynamic operational analysis, and long-term reliability assessments. These advancements, coupled with physical prototype testing, will bridge the gap between computational design generation and practical application, ultimately leading to validated high-performance TES tanks for commercial adoption.

To further enhance the applicability of AI-driven biomimetic TES tank designs, future research should investigate strategies for scaling the optimized structures to industrial applications. While the generated designs closely resembled experimentally validated configurations, modifications would be required to ensure performance consistency in large-scale systems. Adapting fabrication techniques, such as metal-based additive manufacturing, could enable structural robustness while maintaining intricate biomimetic geometries. Additionally, integrating CFD simulations with experimental validation would help assess the impact of varying flow rates, pressure drops, and turbulence characteristics on thermal efficiency. These steps would refine the AI-generated designs and facilitate their practical implementation in real-world energy storage systems.

## 6. Conclusions

This study demonstrated the effectiveness of combining biomimetic design principles with genetic algorithm optimization to enhance thermal energy storage tank performance. The research successfully developed and implemented a systematic optimization framework that leveraged natural thermal distribution patterns, particularly the branching structures found in trees and vascular systems, to improve TES tank design. Through careful implementation of GA optimization, the bio-inspired TES tank design achieved a significant 29% increase in heat transfer surface area within constrained physical dimensions, validating the potential of this novel approach while maintaining the fixed shell dimensions of 150 mm diameter and 155 mm height, ensuring manufacturability.

The optimization framework effectively balanced multiple design parameters, such as branching angles, pipe diameters, and hierarchical structures, while adhering to practical manufacturing constraints. The study revealed distinct evolutionary phases of the optimization process, showcasing the ability of GA to refine designs systematically from exploration to convergence.

The optimization process showcased distinct phases, progressing from rapid initial improvements to systematic convergence, while balancing multiple design parameters, including branching angles, tube diameters, and hierarchical structures. This robust framework successfully integrated biomimetic principles with AI-driven optimization, addressing a critical gap in TES design methodologies.

By establishing a scalable and adaptable optimization approach, this work advances TES technology, offering immediate performance enhancements and laying a strong foundation for sustainable energy storage solutions. The results demonstrated the potential of combining natural design templates with computational intelligence to revolutionize energy storage system development.

## Figures and Tables

**Table 1:** Application of GA-biomimetic integration in various fields.

Field	Application	Summary	Reference
Structural engineering	Optimization of building designs for energy efficiency and material usage.	Utilized GA to optimize building thermal designs, achieving significant energy savings.	Ferdyn-Geygierek et al. (2017) [28]
Aerodynamics	Design of biomimetic airfoil structures.	Applied GA to develop airfoil shapes inspired by bird wings, enhancing aerodynamic performance.	Lu et al. (2021) [29]
Robotics	Development of adaptive robotic systems.	Utilized a genetic algorithm to compute fast, robust, and efficient solutions for target distribution and sequencing in robotic systems.	Valero-Gomez et al. (2011) [30]
Material science	Design of advanced composite materials.	Employed GA to optimize composite material structures inspired by natural materials, enhancing strength and flexibility.	Winkler (2017) [31]
Control systems	Optimization of control strategies in engineering systems.	Applied GA to develop control algorithms inspired by natural processes, improving system stability and performance.	Li et al. (1995) [32]
Automotive engineering	Design optimization of vehicle components.	Applied a biomimetic optimization design to the stiffeners layout of automotive inner door panels, inspired by the vein unit of a dragonfly wing, resulting in improved structural performance.	Xu et al. (2021) [33]
Aerospace engineering	Optimization of spacecraft structures.	Applied GA to design lightweight and robust spacecraft components inspired by biological forms.	Benken et al. (2023) [34]
TES tank design	Application of GA-biomimetic methodology for optimizing TES tank design.	This study introduces a novel integration of GA and biomimetic principles to enhance TES tank performance.	This study

**Table 2:** Summary of key studies on TES optimization and biomimetic approaches.

Reference	Focus Area	Key Contribution	Relevance to the Study
Jouhara et al. (2020) [36]	TES fundamentals	Comprehensive review of TES systems and material developments	Provides foundational knowledge of TES technology
Cabeza et al. (2021) [3]	TES fundamentals	Highlighted thermal conductivity enhancements of up to 85% through material and system integration	Establishes baseline for TES system improvements
Yang et al. (2021) [22]	Shell-and-tube TES design optimization	Achieved 40% reduction in charging time through optimized material selection and geometry	Demonstrates the effectiveness of geometric optimization in TES design
Bouchenna et al. (2021) [38]	TES tank geometry optimization	Enhanced heat transfer rates by up to 55% with optimized configurations	Shows correlation between geometric design and thermal performance
Garcia-Torres et al. (2021) [45]	Stochastic optimization for hybrid energy storage	Applied stochastic optimization techniques for hybrid renewable energy systems with electric and thermal storage integration	Demonstrates stochastic methods for optimizing energy storage strategies
Jamei et al. (2021) [50]	Biomimetic TES design	Documented energy efficiency enhancements of up to 45% in building applications	Validates biomimetic design for practical TES applications
Huang et al. (2023) [41]	Biomimetic fins for heat recovery	Enhanced phase-change heattransfer processes for data centers	Demonstrates application of biomimetic principles for industrial systems
Kumar et al. (2023) [43]	Nano-enhanced PCMs	Achieved 127% improvement in thermal conductivity	Provides state-of-the-art material solutions for TES
Zhang et al. (2023) [39]	Biomimetic TES design	Demonstrated heat transfer improvements of 40–60% through bio-inspired geometries	Highlights effectiveness of biomimetic forms in TES
Zhou et al. (2024) [44]	Thermochromic microencapsulated PCMs	Developed leak-proof reversible thermochromic microcapsule PCMs with high latent heat storage capacity and excellent thermal stability	Provides advancements in material-based TES solutions
Omidvarnia et al. (2024) [25]	Biomimetic TES design	Improved thermal distribution efficiency by 35–50% using vascular-inspired designs	Validates the potential of biomimetic principles in TES optimization
Mohtasim and Das (2024) [40]	Biomimetic composite PCMs	Achieved significant thermal conductivity enhancements in TES applications	Highlights advancements in bio-derived materials for TES
Liu et al. (2024) [64]	Advanced TES materials	Developed phase change materials with up to 52% energy density improvement	Focuses on material innovations to complement design optimization
Xu et al. (2024) [58]	Hybrid optimization for heat exchangers	Combined NSGA-II and IWO to achieve a 25% improvement in thermal performance	Demonstrates the power of hybrid AI approaches in thermal system optimization
Shokouhmand et al. (2020) [59]	Multi-objective optimization of plate-fin heat exchangers	Minimized flow maldistribution and maximized thermal efficiency	Demonstrates effectiveness of multi-objective optimization in heat exchanger design.
Sigmund and Maute (2013) [46]	Topology optimization	Comprehensive review of topology optimization for improved thermal and flow efficiency	Validates the structural efficiency benefits of topology optimization in heat exchangers
Fernández-Godino et al. (2016) [47]	Multi-fidelity optimization frameworks	Reviewed integration of low- and high-fidelity simulations for computational efficiency	Highlights cost-effective modeling approaches for TES optimization
Thuerey et al. (2020) [48]	Deep learning for fluid dynamics optimization	Applied CNNs for heat transfer prediction, reducing computational time significantly	Highlights potential of deep learning for TES optimization
Olabi et al. (2023) [52]	AI-driven TES optimization	Demonstrated performance improvements of 25–40% through AI-optimized thermal storage systems	Establishes AI techniques for TES system enhancement
Moon et al. (2021) [61]	Ultra-power-dense heat exchangers	Achieved 203% higher specific power using genetic algorithms and additive manufacturing	Highlights thecapability of GA in advancing manufacturable designs.
Makhadmeh et al. (2022) [60]	Multi-objective hybrid algorithms for TES	Reviewed advancements in multi-objective hybrid algorithms for thermal energy systems	Supports need for advanced hybrid techniques
Colaço et al. (2022) [62]	Hybrid optimization for double-pipe heat exchangers	Improved thermal performance index by 78% through RL-based genetic algorithms	Demonstrates the effectiveness of hybrid optimization techniques.
Current Study	AI-driven biomimetic TES design generation	Generated novel TES tank designs that resemble experimentally validated structures, achieving a 29% improvement in heat transfer surface area while ensuring manufacturability	Introduced a new methodology that integrates AI-driven generative design with biomimetic principles, moving beyond conventional TES optimization approaches.

**Table 3:** Dimensions of the original bio-inspired TES tank design from Cabeza et al. (2024) [11] used as baseline for optimization.

Shell Measurements (Outer Surface)	
Shell diameter	150 mm
Shell height	155 mm
Tube Measurements (Outer Surface)	
Parent pipe diameter	11 mm
Final parent diameter after tapering at level 0	8 mm
Children after tapering at level 1	6 mm
Parent pipe thickness	0.65 mm
Angle between the parent pipe and children	59°
Angle between the parent pipe and grandchildren	59°
Total number of branches at level 0	7
Total number of branches at level 1	49
Total surface area of tubes	6.6 × 10^4^ mm^2^

**Table 4:** Design parameter bounds for optimization.

Parameter	Lower Bound	Upper Bound	Units
Parent length	20	45	mm
Parent diameter	8	13	mm
Final parent diameter	8	11	mm
Number of branches at level 0	2	10	-
Branching angle at level 0	30	70	Degrees
Children pipe length at level 1	10	30	mm
Children pipe diameter	6	9	mm
Final diameter of children pipe	6	9	mm
Grandchildren pipe length at level 2	10	20	mm
Grandchildren pipe diameter at level 2	6	9	mm
Final diameter of grandchildren pipes	4	6	mm
Number of branches at level 1	7	64	-
Branching angle at level 1	30	70	Degrees

**Table 5:** GA implementation parameters.

Parameter	Size
Population size	100
Crossover probability	0.3
Mutation probability	0.7
Individual probability distribution	0.5
Distribution index	39
Number of generations	500
Tournament size	3

**Table 6:** Optimization process parameters and controls.

Component	Parameter	Value/Method
Selection	Primary method	Roulette wheel
	Secondary method	Tournament selection
	Tournament size	3
Evolution control	Initial population	Random feasible design
	Population size	100
	Generation limit	500
	Convergence check	Every 10 generations
Genetic operators	Crossover type	One-point
	Crossover rate	Adaptive (0.3–0.7)
	Mutation type	Bit-flip
	Mutation rate	Adaptive (0.1–0.3)

**Table 7:** Validation criteria and methods.

Validation Level	Parameters Checked	Verification Method
Geometric	Shell dimensions	CAD model verification
	Tube spacing	Minimum distance check
	Branch angles	Angular constraints
Performance	Surface area	Analytical calculation
	Heat transfer	Theoretical models
	Manufacturing	Design rule check
Convergence	Fitness progress	Generation-wise analysis
	Population diversity	Statistical measures
	Solution quality	Comparative assessment

**Table 8:** Best design parameters concerning optimization of input parameters with a fixed range of value.

Input Parameters	Best Individual Output
Parent length	45 mm
Parent diameter	13 mm
Final parent diameter	11 mm
Children pipe length	30 mm
Children pipe diameter	9 mm
Final diameter children pipe	8 mm
Grandchildren length	20 mm
Grandchildren diameter	8 mm
Final diameter grandchildren	6 mm
Number of branches at level 0	8
Angle at level 0	50°
Number of branches at 1	64
Angle at level 1	50°

**Table 9:** Comparison between design parameters of preliminary prototype and GA-optimized design.

Parameters	Prototype Dimensions	Simulated/ Optimized Dimensions
Parent pipe diameter	11 mm	13 mm
Parent pipe diameter after tapering at level 0	8 mm	11 mm
Children pipe diameter after tapering at level 1	6 mm	7 mm
Angle between parent pipe and level 0 branches	59°	50°
Angle between children pipe and level 1 branches	59°	50°
Total heat transfer area	6.6 × 10^4^ mm^2^	7.9 × 10^4^ mm^2^
Total number of branches at level 0	7	8
Total number of branches at level 1	49	64

**Table 10:** Comparative analysis of the proposed method with existing studies.

Parameters	Approach	Performance Improvement	Key Features	Limitations
This Study	GA + Biomimetic Design	29% surface area improvement	First AI + biomimicry-based generative design framework for TES tanks; systematically generates bio-inspired structures while maintaining manufacturability constraints	No experimental validation yet
Zhang et al. (2023) [39]	Biomimetic oval geometries	40–60% heat transfer improvement	Application-specific heat storage designs	No optimization, limited generalization
Huang et al. (2023) [41]	Biomimetic fins	Improved PCM heat transfer	Waste heat recovery focus, biomimetic fin structures	Limited scalability for other TES designs
Mohtasim and Das (2024) [40]	Bio-derived PCMs	Increased thermal conductivity	Material-based improvements for TES	No geometric or structural optimization

**Table 11:** Diversity matrix for GA optimization.

Generation	Standard Deviation (mm^2^)	Coefficient of Variation (%)
0	12,378.00	54.5
50	9561.13	12.8
200	10,405.80	13.9
500	11,777.90	15.8

**Table 12:** Computational cost assessment.

Optimization Method	Computational Cost (s)	Population Size	Generations
Genetic algorithm (Current Study)	3.51	100	500
Particle swarm optimization (PSO) [73]	5.23	100	500
Simulated annealing (SA) [74]	6.47	N/A	Iterative

## Data Availability

Data available under request to the correspondence authors.

## Abbreviations

The following abbreviations are used in this manuscript:

abbreviations	
TES	Thermal energy storage
GA	Genetic algorithm
AI	Artificial intelligence
CAD	Computer aided design
Fusion 360	Software used for designing the bio-inspired TES tank prototype
Python 3.1.2.3	Programming language version used for the GA development
DEAP	Distributed evolutionary algorithms in Python, a framework used for the implementation of GA
NSGA-II	Non-dominated sorting genetic algorithm II
MOPSO	Multi-objective particle swarm optimization
Symbols	
$P_{d}$	Parent pipe diameter
$P_{r1}$	Parent pipe radius
$Fp_{d}$	Final parent pipe diameter after tapering
$P_{r2}$	Final parent pipe radius after tapering
$C_{d}$	Children pipe diameter
$C_{r1}$	Children pipe radius
$Fc_{d}$	Final children pipe diameter after tapering
$C_{r2}$	Final children pipe radius after tapering
$Gc_{d}$	Grandchildren pipe diameter
$Gc_{r1}$	Grandchildren pipe radius
$FGc_{d}$	Final diameter of grandchildren pipe after tapering
$Gc_{r2}$	Final grandchildren pipe radius after tapering
$S_{P}$	Slant height of a parent pipe
$S_{C}$	Slant height of a children pipe
$S_{GC}$	Slant height of a grandchildren pipe
$P_{length}$	Parent pipe length
$C_{length}$	Children pipe length
$Gc_{length}$	Grandchildren pipe length
$A_{P}$	Area of a parent pipe
$A_{C}$	Area of a children pipe
$A_{GC}$	Area of a grandchildren pipe
$A_{T}$	Total surface area
$n_{b0}$	Number of branches at level 0
$n_{b1}\;$	Number of branches at level 1

## Appendix A. Genetic Algorithm Implementation for Bio-Inspired TES Tank Optimization

The following steps outline the algorithm implemented in the code used for this manuscript.

Define parameters and problem: • **Set constants** for the shell dimensions: ○ Shell Length (SHELL_LENGTH) and Shell Diameter (SHELL_DIAMETER). • **Define the fitness function** (fitness) to maximize the total exposed surface area of the structure. Fitness function: • **Input**: Genotype of an individual parameters. • **Calculation**: ○ Compute radii, slant heights, and surface areas for: ▪ Parent pipe. ▪ Children branches. ▪ Grandchildren branches. ○ Sum all surface areas to derive the fitness value (aimed to maximize surface area). Constraint function: • Ensure that the total structure length does not exceed the **shell length**. • Ensure that the diameter does not exceed the **shell diameter**. • Return True if constraints are satisfied; otherwise, return False. Setup genetic algorithm components: • Use the **DEAP library** to define: ○ **Fitness type**: Miaximize fitness value. ○ **Individual representation**: A list of numeric parameters. ○ **Population**: A collection of individuals. Attribute initialization: • Define parameter ranges for the genotype using random sampling techniques (e.g., uniform distribution, random integers). Individual and population creation: • Use tools.initCycle to assemble an individual with the defined attributes. • Generate a population by repeating the individual creation process. Penalty for infeasible individuals: • Apply a penalty function using tools.DeltaPenalty for individuals that violate constraints. Define genetic operators: • **Evaluation**: Apply the fitness function. • **Crossover**: Perform two-point crossover. • **Mutation**: Use uniform mutation with bounds for each parameter. • **Selection**: Apply tournament selection with a tournament size of 3. Run the genetic algorithm: • **Initialize the population** and define statistics tracking. • Use algorithms.eaSimple to perform the evolutionary process: ○ **Crossover Probability**: 0.3 ○ **Mutation Probability**: 0.7 ○ **Number of Generations**: 500 • Track the evolution of population and update statistics. Store and analyze results: • Retrieve the best individual using HallOfFame. • Compute and display the **total surface area** of the best solution. • Plot the fitness statistics over generations. Visualization: • Generate a graph to visualize the evolution of fitness across generations. Main execution: • Seed the random number generator for reproducibility. • Execute the algorithm and display the results.
